# Realization of Forest Internet of Things Using Wireless Network Communication Technology of Low-Power Wide-Area Network

**DOI:** 10.3390/s23104809

**Published:** 2023-05-16

**Authors:** Ming Zhao, Ren-Jie Ye, Shuo-Tsung Chen, Yen-Chun Chen, Zi-Yu Chen

**Affiliations:** 1 School of Computer Science, Yangtze University, Jingzhou 434023, China; 2 Graduate School of Applied Chinese Studies, National Yunlin University of Science and Technology, Douliu 64002, Taiwan; 3 Department of Medical Informatics, Chung Shan Medical University, Taichung 40201, Taiwan; 4 Department of Information Center, Chung Shan Medical University Hospital, Taichung 40201, Taiwan

**Keywords:** intelligent forest monitoring, Internet of things (IoT), low-power wide-area network (LPWAN), long range (LoRa), narrow-band Internet of things (NB-IoT)

## Abstract

This work implements an intelligent forest monitoring system using the Internet of things (IoT) with the wireless network communication technology of a low-power wide-area network (LPWAN), a long range (LoRa), and a narrow-band Internet of things (NB-IoT). A solar micro-weather station with LoRa-based sensors and communications was built to monitor the forest status and information such as the light intensity, air pressure, ultraviolet intensity, CO_2_, etc. Moreover, a multi-hop algorithm for the LoRa-based sensors and communications is proposed to solve the problem of long-distance communication without 3G/4G. For the forest without electricity, we installed solar panels to supply electricity for the sensors and other equipment. In order to avoid the problem of insufficient solar panels due to insufficient sunlight in the forest, we also connected each solar panel to a battery to store electricity. The experimental results show the implementation of the proposed method and its performance.

## 1. Introduction

In recent years, the concept of the Internet of things (IoT) has developed rapidly and has been applied successfully in different fields, including city applications [1,2,3,4,5,6,7,8], medical applications [9,10,11,12,13,14,15,16,17,18], industrial applications [19,20,21,22], agricultural applications [23,24,25,26,27], and forestry applications [28,29,30,31,32,33,34,35,36,37,38,39,40,41,42,43]. The applications using IoT technology in forestry can be classified into three major aspects: forest resource planning and environmental monitoring, the intelligent management of forest fires, and the prevention of illegal logging. Forestry IoT technology has been successfully applied to forest resource planning and environmental monitoring using a wireless sensing network (WSN). Zhang et al. [28] proposed a platform of forest environmental factor collection based on the ZigBee protocol. The platform includes many different types of terminal monitoring equipment such as temperature, humidity, water level, gas, micro-electromechanical system (MEMS), photoresistor, and human infrared sensors. Anitha and Ravi [29] discussed the WSN-based landslide forecasting and geographical information system as an overview. Suciu et al. [30] used available resources to present an effective WSN architecture with a solution. In the WSN architecture, they tried to optimize the duty cycle of a single sensor (but a single node) or maximize the network service life. Ganesh et al. [31] proposed an IoT-based environmental monitoring system using a WSN.

Next, we reviewed studies related to intelligent forest fire management. In [32,33,34], the authors proposed a forest fire detection system that randomly deployed sensor nodes in the forest area, with each node being equipped with a temperature sensor. Moreover, each node checked the environment periodically to determine whether there was an emergency. When some sensor nodes detect a significant change in temperature, they will broadcast data packets containing the measured values. At the same time, the measured values are presented on computer web pages and mobile phone pages. Generally speaking, the higher the sensor density, the more reliable the data obtained. However, saving energy is also one of the important issues in WSNs. There is a compromise between the two. Therefore, Ma et al. [35] proposed a mathematical solution called the forest fire monitoring paradigm (FFMP). The purpose of the FFMP is to provide early detection and location information for forest fires based on a hybrid WSN. Differently from pure static and mobile WSNs, an FFMP hybrid WSN is composed of mobile sensor nodes and static sensor nodes. Hybrid wireless sensor networks are a compromise between cost and coverage. The system proposed by Chandrasekharan et al. [36] consists of temperature sensors and a GSM module, where the temperature sensors use tree power for their operation and the GSM module is connected to the GSM network to transmit the detected fire alarm signal.

In illegal logging prevention, the sensor network by Miroslav et al. [37], which uses sound collection and actual recording, is the first anti-pilot logging system that can continuously provide real-time monitoring for endangered forests. Sarde and Kshirsagar [38] recorded the sound through a microphone and loaded the sound into matlab software on the network to perform a Fourier transform operation to transform the time domain into the frequency domain for analysis and recognition. Narhari R. Kotkar [39] developed a system that can be used to stop smuggling. Each tree is equipped with a small electronic device composed of a microcontroller, a flexible vibration sensor, and a Zigbee module. The flexible vibration sensor detects tree cutting and the communication is completed by the Zigbee, GSM-modem, CMGF-message-format module. The smuggling/theft of important trees such as sandalwoods in the forest poses a serious threat to forest resources, causes significant economic losses, and ultimately causes considerable destructive effects on the environment around the world. Therefore, in the literature [40,41], the authors used MEMS technology and the forest sector to use renewable solar energy vibration sensors to monitor special trees such as sandalwoods. Suguvanam et al. [42] provided a method that included three units, a tree unit, a sub-server unit, and a forest officer, to prevent the smuggling of valuable trees such as sandalwoods and red sandalwoods in forest areas. In the tree unit vibration sensor, the sensor is deployed using continuity. An RFID receiver and a ZigBee transmitter are connected to the microcontroller. One subserver was used for every 50 trees. In the subserver, GPS, GSM, and ZigBee receivers are connected.

In the above concept, the information collected by deploying various traditional sensors and types of monitoring equipment can be received through different wireless communication methods such as Zigbee, WiFi, 2G, 3G, or 4G. However, they are all limited by transmission distance, power consumption, and transmission costs. The aim of this work is to implement an intelligent forest Internet of things (IoT) using the wireless network communication technology of a low-power wide-area network (LPWAN). The proposed contributions include the following: 1. We selected two LPWAN technologies; long range (LoRa), which uses unlicensed frequency bands, and narrow-band Internet of things (NB-IoT), which uses licensed frequency bands, combined with their respective sensors, including a LoRa-support solar micro-weather station and NB-IoT-support sensors. 2. A multi-hop technique for the LoRa-based sensors and communications was proposed to solve the problem of long-distance communication without 3G/4G. 3. For the forest without electricity, we installed solar panels to supply electricity to the sensors and other equipment. In order to avoid the problem of insufficient solar panels due to insufficient sunlight in the forest, we also connected each solar panel to a battery to store electricity. Thus, sensing data on parameters such as the light intensity, air pressure, ultraviolet intensity, and CO_2_ were transmitted successfully to a LoRa gateway connected to 3G/4G. Finally, the real-time sensing data were transmitted to a web server.

The rest of this work is organized as follows. Section 2 provides the background related to the forest sensors and communication technology proposed in Section 3. The proposed forest sensors and communication technology are presented in Section 3. Section 4 shows the implementation and experimental results. Section 5 gives a conclusion.

## 2. Background and Related Work of LPWAN

### 2.1. Background

**Figure 1:**
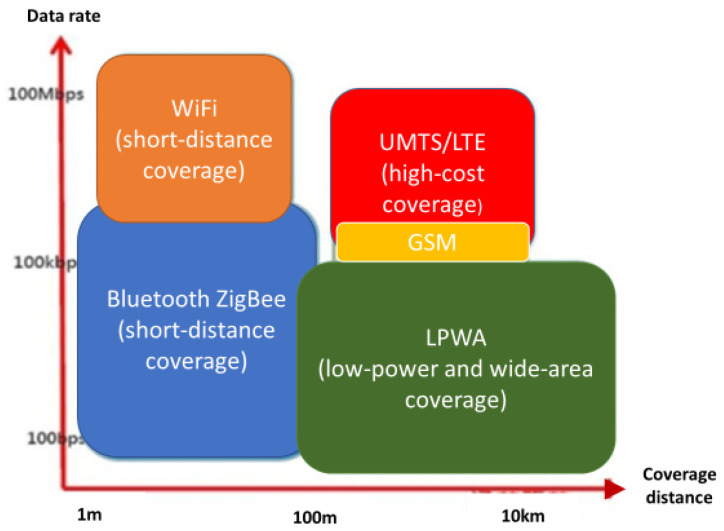
Comparison of LPWAN and other communication technologies.

In the past, the information collected by various monitoring equipment could be used through different wireless communication methods such as Zigbee, WiFi, 2G, 3G, or 4G. However, they were all limited by the transmission distance, power consumption, and transmission costs and could not be deployed in a large area, especially in a forest area. For long-distance, low-power, high-coverage wireless communication needs, the newly developed wireless network communication technology of an LPWAN in recent years can be roughly divided into two categories: an NB-IoT, which uses licensed frequency bands, and LoRa, Sigfox, and Weightless, which use unlicensed frequency bands. As shown in Figure 1, compared with other communication technologies, an LPWAN has many advantages, such as its long-distance range, high coverage, low power consumption, low cost, and so on. At present, the global Internet of things and communication manufacturers have actively set up various cross-domain or independent LPWAN base stations, combining their advantages of a long-distance range, high coverage, low power consumption, and low cost with related sensors and smart devices to provide related innovative services. In addition to the common characteristics of a long-distance range, low power consumption, and high coverage, this section evaluates and analyzes the advantages and disadvantages of the above-mentioned LPWAN communication technology and its application areas as follows:

NB-IoT: An NB-IoT is a carrier-grade network supported by the international telecommunications standards development organization, 3GPP, and built for the IoT. It has a good network transmission quality, high data security, and a low construction cost. Based on the low-rate IoT market in the licensed spectrum, an NB-IoT can be deployed directly on LTE networks, and an NB-IoT can be quickly deployed without drastically changing the current 4G LTE telecommunication network architecture. In addition, an NB-IoT can also be deployed through equipment upgrades based on the current operator’s existing 2G, 3G, and 4G networks, which can reduce deployment costs. As a result, an NB-IoT is supported by telecom companies in various countries and is a widely used Internet-of-things technology worldwide. The Global Mobile Suppliers Association announced in March 2019 that more than 100 operators have deployed an NB-IoT.

LoRa: LoRa is the basis of the wireless communication technology developed by the American semiconductor manufacturer Semtech through its acquisition of the French company Cycleo, with the specification completed in cooperation with IBM. The LoRa Alliance, composed of Semtech, IBM, and Cisco as the core, promotes related development. LoRa, like Wi-Fi, adopts a mode in which anyone can set up a Wi-Fi access point to build a network environment. With more than 500 members, it can be said to be the most industry-supported LPWAN technology.

LoRa’s communication protocol and network architecture will directly affect the battery life, network capacity, service quality, security, and network applications of the node. LoRa is a technology that modulates data into electromagnetic waves. The transmission method used is called the “chirp spread spectrum”. LoRa nodes can only be used to transmit data. Supporting two-way communication is one of the main features of LoRa, which allows terminal devices such as sensors to send information such as the location, occupancy, and data to the network, and receive messages from the network.

Sigfox: Although Sigfox from France uses an unlicensed spectrum, it needs to rely on base stations built by manufacturers in order to strike a balance between cost and communication quality. Sigfox is the technology with the lowest transfer rate; the speed is only 100 bits/s, each device can only transmit 140 messages a day, and the maximum capacity of each message is 12 bytes. As the amount of information transmission is reduced, the power consumption of IoT devices can be greatly reduced. For example, applications such as electricity meters, water meters, and street lamp controls are suitable for Sigfox because the flow rate reporting frequency is less than once per hour. In addition, Sigfox also provides SigfoxCloud cloud system integration services, which can reduce the complexity of users developing programs and accessing the data. At present, Sigfox has promoted the deployment of base stations in 32 countries around the world, including France, the United Kingdom, Spain, Germany, the Netherlands, and the United States.

Weightless: The Weightless Technology and Standards Organization was founded by Dr. William Webb in 2012. The initial board members included Neul, ARM, Cable & Wireless, CSR, and other companies. Dr. William Webb serves as the CEO of the Weightless SIG Standards Organization, and concurrently serves as the co-founder and CTO of Neul. The Weightless standard was released in April 2013. In response to the needs of the IoT communication market, in accordance with the goal of city-level networking, the Weightless Internet-of-things wireless communication standard was created.

Weightless has three different architectures: Weightless-N, Weightless-P, and Weightless-W. Weightless-W is the earliest developed version, which mainly uses TV white space (TVWS) to transmit data. However, due to the significant differences in the development process of TVWS released by various countries, its development is limited. The Weightless-N version adopts a sub-GHz license-free frequency band; the transmission rate is 30~100 kbit/s and the transmission distance is about 5 km. Weightless-W and Weightless-N only support one-way transmission, while the latest version, Weightless-P, supports two-way communication, and its transmission rate and transmission distance are about 100 kbit/s and up to 2 km, respectively.

As shown in Table 1, under the same conditions, LoRa has the best network signal coverage, but in terms of the number of supported nodes and spectrum efficiency, Weightless performs better than LoRa and Sigfox.

### 2.2. Related Work to Applications of LPWAN

In this sub-section, we summarize the current solutions based on similar networks in Section 2.1 for forests or environmental monitoring. In Refs. [43,44], data communication networks that can inform about forest fires in real-time were developed that could use long-distance, wireless-communication-based tools, namely LoRa. In terms of data transmission, the use of LoRa as an early warning method for monitoring forest fires in the Riau province allowed data to be sent from sensors to a gateway as far as 30 miles away [45]. In Ref. [46], M. I. Nashiruddin and S. Winalisa designed a LoRaWAN Internet-of-things network for Smart Manufacture on Batam Island. LoRa acquires high recognition by providing a low cost for IoT modules and equipment, M2M, and other industrial needs [47]. In order to monitor the system conditions perfectly, the authors of Ref. [48] incorporated LoRaWan-based IoT technology into the smart field environment. In Ref. [49], the authors applied IoT technology to propose a novel forestry management system in the Fushan Botanical Garden in Taiwan. Their system techniques included the following: 1. Transmission of sensing data on forest information using the wireless network communication technology of a low-power wide-area network (LPWAN) such as LoRa or an NB-IoT. 2. Application of different sensing techniques to process forest resources and monitor the microclimate changes in a forest. In Ref. [50], the authors presented the design and implementation of a LoRa-based forest-fire-monitoring system. The LoRa-based forest-fire-monitoring system technology was based on wireless technology that can transmit data across the forest. To detect the presence of a fire, Arduino Uno was used as a microcontroller that regulates the input from the AMG8833 sensor and GPS Ubox 6M. In Ref. [51], the authors built a LoRa-based smart agricultural management and monitoring system using a WSN in rural areas to replace the current technology of the agricultural monitoring system. In Ref. [52], the authors proposed the design and implementation of a LoRa-based wireless sensor network for monitoring the quality of water in coastal areas, rivers, and ditches with the aim of generating an observatory of water quality for the monitored areas. A comparison between the proposed solution with other methods is listed in Table 2.

## 3. Proposed Forest Sensors and Communication Technology

**Figure 2:**
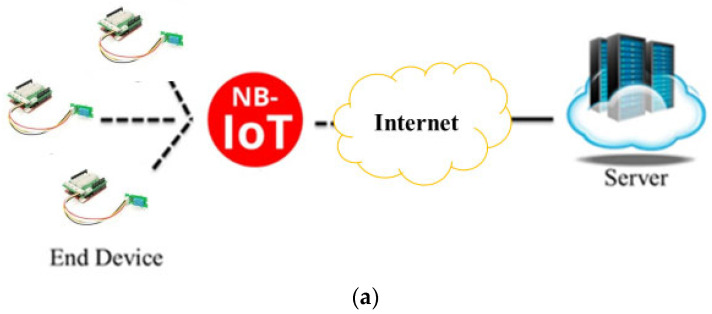

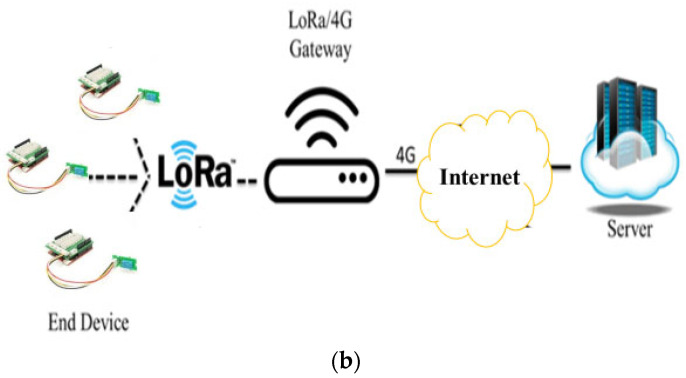
Two communication technologies, NB-IoT and LoRa, combined with their respective sensors. (**a**) NB-IoT communication technology and its respective sensors. (**b**) LoRa communication technology and its respective sensors.

Based on the discussion in the last section, we adopted the two wireless network communication technologies LPWAN and NB-IoT, which use licensed frequency bands, and LoRa, which uses unlicensed frequency bands, combined with their respective sensors, as shown in Figure 2, to be the proposed forest sensors and communication technology. In forests or mountains, an NB-IoT is used in a small number of areas with 3G/4G telecommunication signals, while RoLa is used mostly in areas without 3G/4G telecommunication signals. The implementation architecture is presented in Section 3.1 and Section 3.2.

### 3.1. NB-IoT Communication Technology and Its Respective Sensors

**Figure 3:**
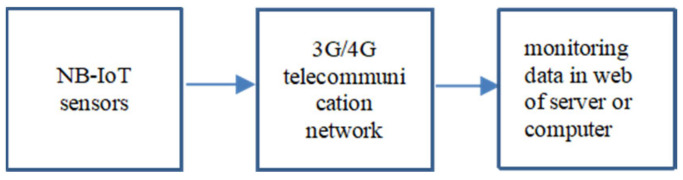
Architecture of NB-IoT communication technology for returning the sensing data.

In a few areas with 3G/4G, we used NB-IoT communication technology to continuously return the sensing data from NB-IoT-support sensors to the server or computer through the 3G/4G telecommunication network. Figure 3 shows the proposed NB-IoT-support sensors and the architecture of NB-IoT communication technology.

### 3.2. LoRa Communication Technology and Its Respective Sensors

**Figure 4:**
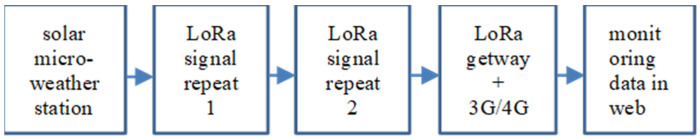
Architecture of LoRa signal repeat method for returning the sensing data of the LoRa-support solar micro-weather station.

**Figure 5:**
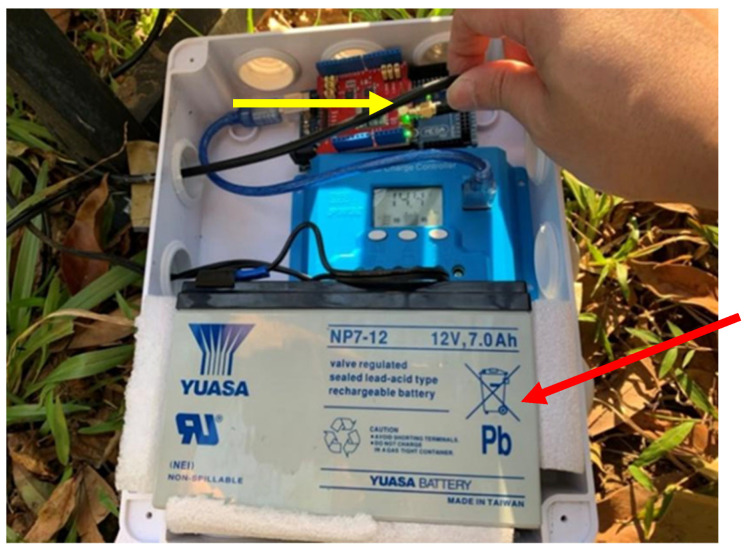
The gateway device (indicated by the yellow arrow) has an external battery (indicated by the red arrow) that can be charged at any time.

In most areas without a 3G/4G telecommunication signal, the proposed LoRa signal repeat method will continuously return the sensing data of the LoRa-support solar micro-weather station. Figure 4 presents the proposed LoRa-support solar micro-weather station and the architecture of the LoRa signal repeat method, which is described in detail as follows. First, the gateway is set up in a place with electricity, such as a street lamp or public facility at the foot of a mountain, and is supplemented by an external battery that can be charged at any time to achieve uninterrupted power and avoid an unstable power supply in mountainous areas, as shown in Figure 5. Next, the LoRa-support solar micro-weather station is set up in a place that needs a forest resource survey or monitoring, and then the related sensing data are transmitted through LoRa communication technology. Whether the transmitted signal strength is sufficient to be received by the gateway is evaluated by the RSSI. The signal strength *p_d_* according to the distance (*d*) can be represented by:

(1) $$p_{d}=p_{0}-10n\log_{10}(d)$$

or:

(2) $$d=10^{\frac{p_{0}-p_{d}}{10n}}$$

where *p*_0_ is the strength of the transmission signal measured at a distance of one meter from the transmitter; *d* is the distance between the transmitter and receiver; and *n* is a signal attenuation constant that can be obtained from the received signal and the actual distance.

Different levels of signal strength can reduce the location errors of the RSSI caused by the interferences. In order to determine the signal repeat location of a specified sensor, we defined the indication function by:

(3) $$I(d)=\left\{\begin{array}{cc} 1, & \mathrm{if}\;d\geq \varepsilon \\ 0, & \mathrm{otherwise} \end{array}\right.$$

where ε denotes the threshold for which the receiver can receive the signal from the transmitter.

If the RSSI determines that the signal strength is not high enough to send the sensing data back to the gateway using (2) and (3), it is necessary to set up a RoLa signal repeat station powered by solar energy. Finally, the gateway sends the received sensing data back to the server or computer through the 3G/4G telecommunication network.

## 4. Experiments and Discussion

This section shows the implementation of the proposed forest monitoring system using the IoT with an LPWAN in the Fushan Botanical Garden in Taiwan. Section 4.1 shows the realization and experimental results of NB-IoT communication in a few areas with a 3G/4G telecommunication signal. Section 4.2 shows the realization and experimental results of LoRa repeat planning for a large area without a 3G/4G telecommunication signal.

### 4.1. Realization and Experimental Results of NB-IoT Communication

**Figure 6:**
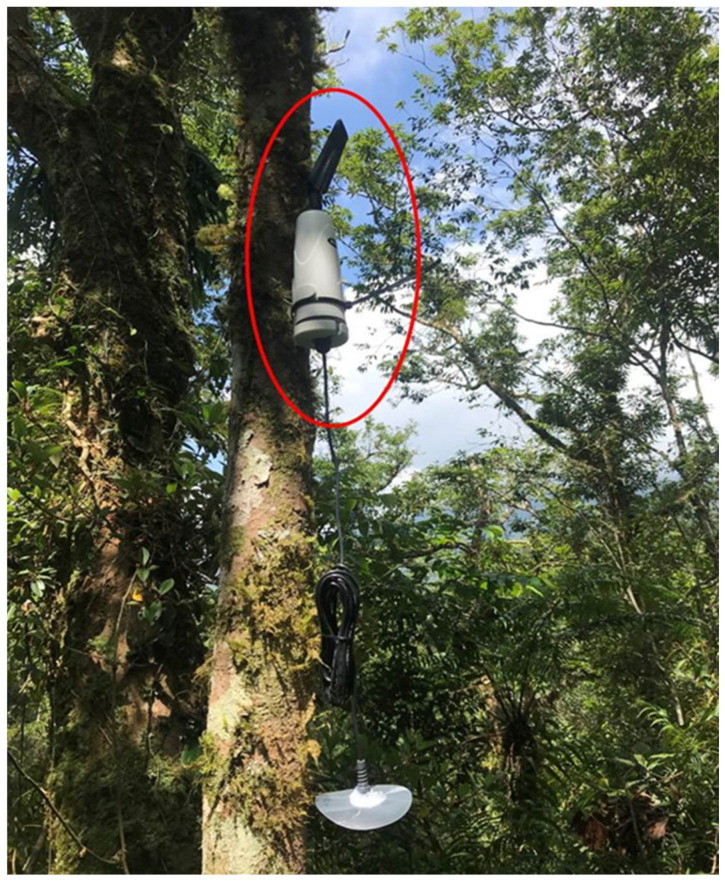
NB-IoT-support temperature and humidity sensors.

**Figure 7:**
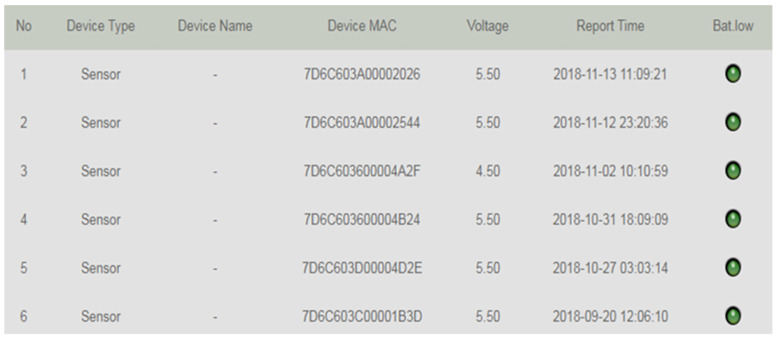
Real-time data on temperature and humidity. Moreover, the green light means the battery has enough power.

In a few areas with a 3G/4G telecommunication signal, as shown in Figure 6, we first set up the NB-IoT-support sensors and continuously sent out their sensing data by using NB-IoT communication technology. Next, the real-time sensing data, including temperature and humidity, were connected to the 3G/4G telecommunication network, and finally, they were transmitted to the web server, as shown in Figure 7.

### 4.2. Realization and Experimental Results of LoRa Repeat Planning for Solving No Signal

**Figure 8:**
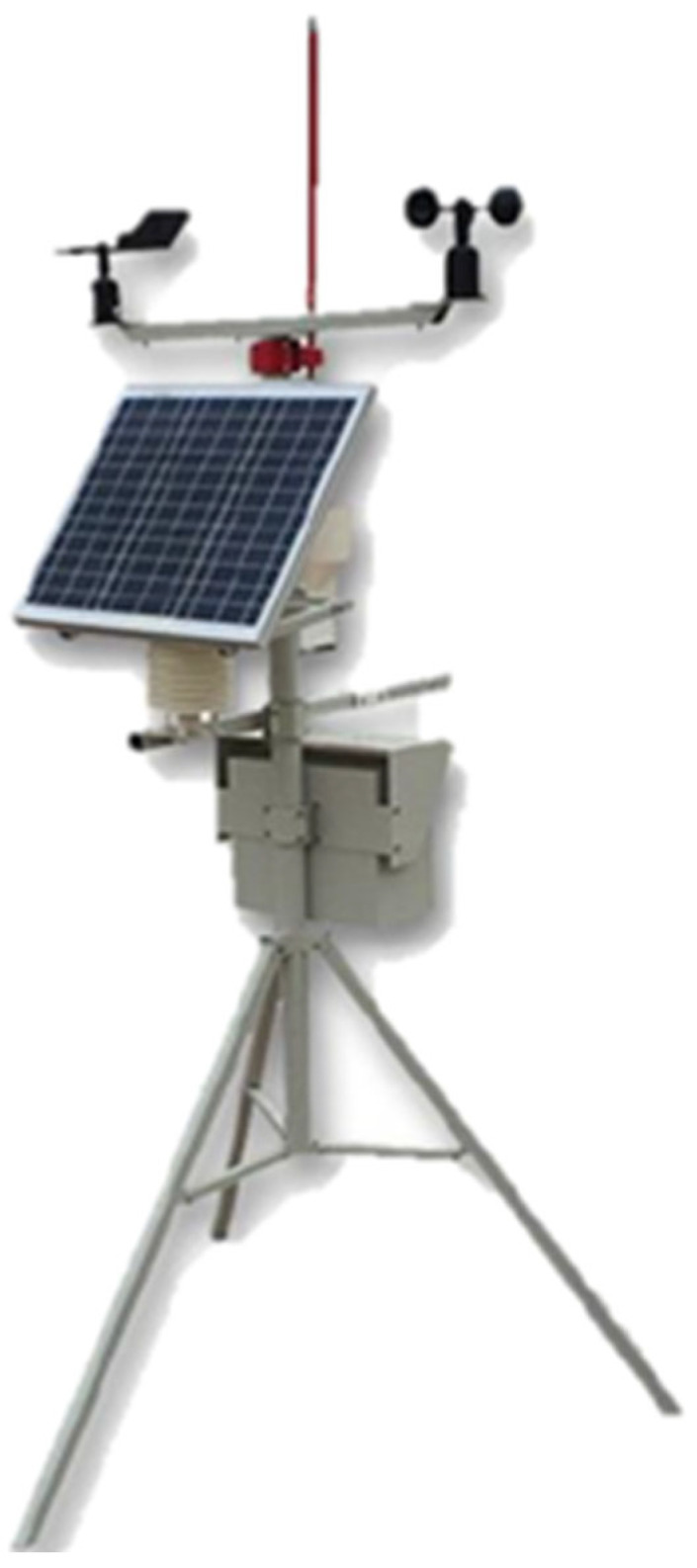
LoRa-support solar micro-weather station.

To transmit sensing data in the large areas without a 3G/4G telecommunication signal, as shown in Figure 8, we first assembled a LoRa-support solar micro-weather station with LoRa communication, an illuminance sensor, an atmospheric pressure sensor, an ultraviolet light sensor, a carbon dioxide sensor, etc. The antenna was 868 megaHz. For convenience, the RSSI values were measured by the tool WifiInfoView. Then, we used the RSSI to measure the signal strength and set up a gateway on the beam of the pavilion at the entrance of the Fushan Botanical Garden, which has both electricity and 3G/4G internet signals. The gateway shared the electricity of the pavilion lights and monitors. In order to avoid an unstable power supply in mountainous areas, the gateway was supplemented by 7A batteries that can be charged at any time to achieve uninterrupted power. Next, we set up a LoRa-support solar micro-weather station near the top of the mountain where we needed to conduct a resource investigation. Thus, the LoRa-support solar micro-weather station could continuously send out sensing data by using LoRa communication technology.

**Figure 9:**
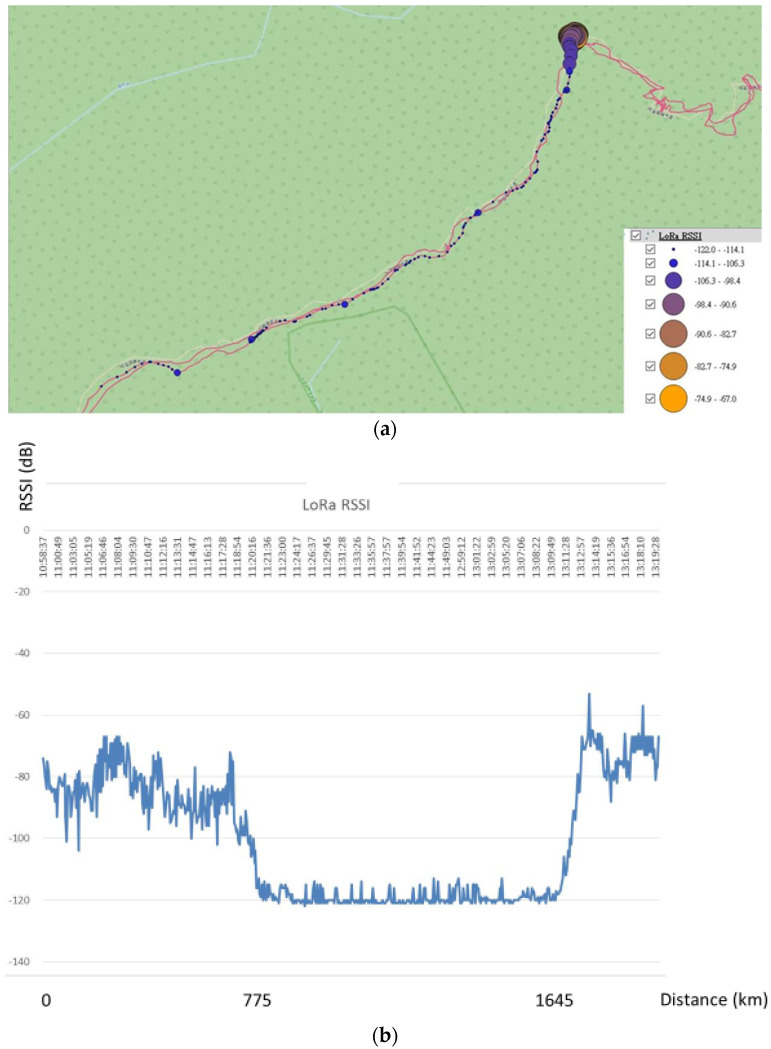
RSSI of LoRa signal between the LoRa-support solar micro-weather station and the gateway. (**a**) The RSSI variety from the LoRa-support solar micro-weather station to the gateway. (**b**) The RSSI from the LoRa-support solar micro-weather station to the gateway.

**Figure 10:**
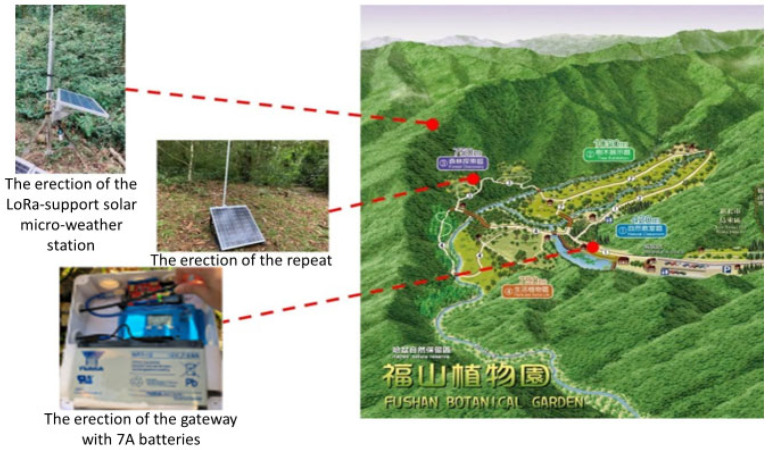
Realization of one LoRa-support solar micro-weather station, one repeat station, and the communication technology.

However, as shown in Figure 9, at a distance of between 775 m and 1645 m from the LoRa-support solar micro-weather station, that is, from 810 m above sea level to 680 m, the signal was lost for an RSSI of −120 dB and was unstable from −110 dB to 120 dB. Therefore, as shown in Figure 10, we initially set up a LoRa repeat station between the LoRa-support solar micro-weather station and the gateway to solve the lost-signal problem so as to successfully repeat the real-time sensing data, including data on the illuminance, atmospheric pressure, ultraviolet light, and carbon dioxide. As shown in Table 3, we evaluated the correspondence between the distance and the signal strength using three times the average of the RSSI from the LoRa-support solar micro-weather station on the mountain slope without rain. In order to reduce the location errors of the RSSI from −110 dB to 120 dB caused by the interference, we excluded the RSSIs below −110 dB, and Equation (3) became: $I(d)=\left\{\begin{array}{cc} 1, & \mathrm{if}\;d\geq 500\\ 0, & \mathrm{otherwise} \end{array}\right.$ where ε=500 m or ε=0.5 km. Then, the sensing data were transmitted to the LoRa gateway, which was connected to the 3G/4G telecommunication network with an average of 10 s. Finally, these real-time sensing (the sensor senses immediately) data were transmitted to a web server within seconds.

**Figure 11:**
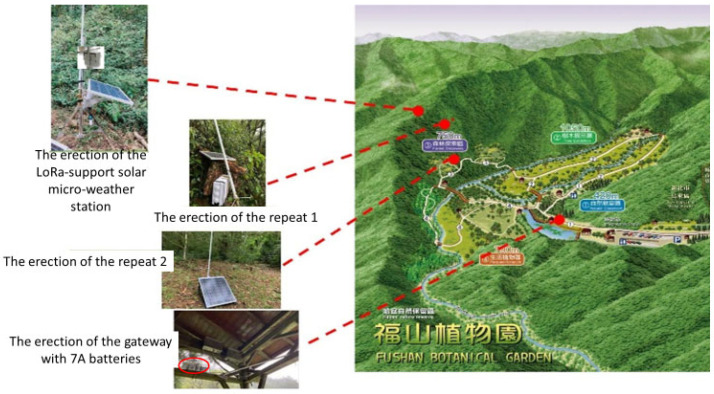
Realization of one LoRa-support solar micro-weather station, two repeat stations, and the communication technology.

**Figure 12:**
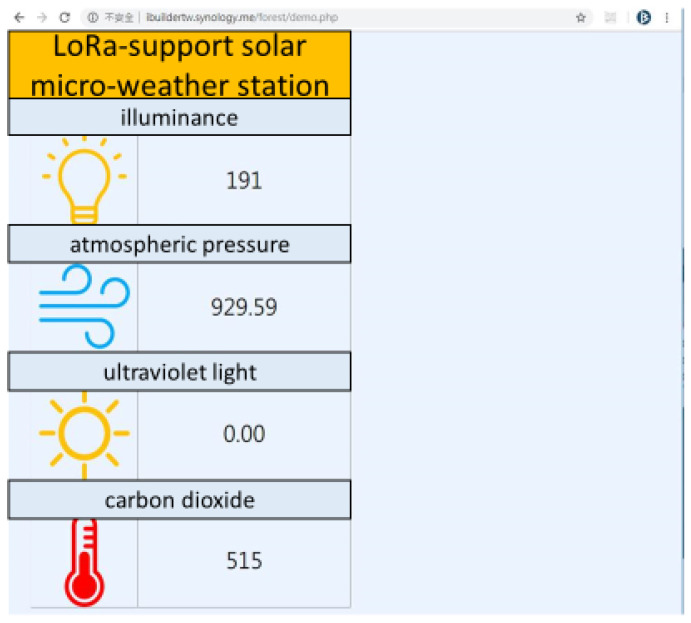
Real-time data on illuminance, atmospheric pressure, ultraviolet light, and carbon dioxide.

**Figure 13:**
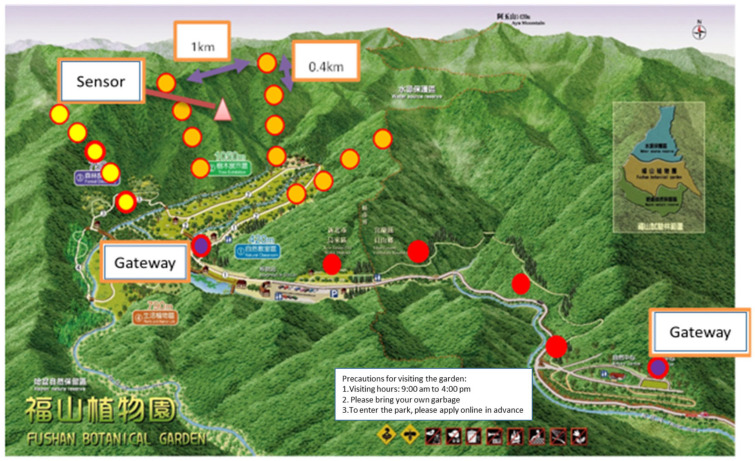
Realization of multiple LoRa-support solar micro-weather stations with repeat stations, and the communication technology.

Furthermore, as shown in Table 4, when it rains heavily, the signal strength was attenuated by about 20% so that Equation (3) became: $I(d)=\left\{\begin{array}{cc} 1, & \mathrm{if}\;d\geq 400\\ 0, & \mathrm{otherwise} \end{array}\right.$ where ε=400 m or ε=0.4 km. Different levels of signal strength can reduce the location errors of the RSSI caused by the interference of heavy rain. Accordingly, we set up a signal repeat station every 0.4 km between the LoRa-support solar micro-weather station and the gateway on the mountain slope. Therefore, we added an additional signal repeat station between the weather station and the original signal repeat station, as shown in Figure 11, to obtain all sensing data in the web server again, as shown in Figure 12. Similarly, we evaluated the correspondence between the distance and the signal strength using three times the average of the RSSI from the LoRa-support solar micro-weather station at the mountain edge without rain in Table 5. Because of the attenuated 20% due to heavy rain, we set up a signal repeat station every 1 km along the mountain edge. Finally, Figure 13 shows the realization of multiple LoRa-support solar micro-weather stations with repeat stations and communication technology.

## 5. Conclusions

In this work, we applied the wireless network communication technology of an LPWAN to implement an intelligent forest Internet-of-things (IoT) management by taking the Fushan Botanical Garden in Taiwan as a real case. In the areas with 3G/4G, we set up NB-IoT-support sensors and continuously sent out their sensing data by using NB-IoT communication technology. In particular, an RSSI multi-hop for the signal repeat of the LoRa-support sensors was proposed to solve the problem of long-distance communication without 3G/4G. For the forest without electricity, we installed solar panels to supply electricity to the sensors and other equipment so that the sensing data on parameters such as light intensity, air pressure, ultraviolet intensity, and CO_2_ would be transmitted to the LoRa gateway. By connecting the LoRa gateway to 3G/4G, the real-time sensing data were successfully transmitted to the web server. In order to avoid the problem of insufficient solar panels due to insufficient sunlight in the forest, we also connected each solar panel to a battery to store electricity.

In the future, we will design a more comprehensive mathematical model that considers the location, number, and cost of weather stations, sensors, relay stations, gateways, and other equipment.

## Figures and Tables

**Table 1:** Main technical comparison table for LPWAN.

Technical Protocol	Promoter	Established Year	Number of Base Station Connections	Frequency Band Used	Transmission Distance	Transmission Speed	Technical Category
Sigfox	Sigfox	2009	1,000,000	ISM band, sub-1 GHz	Urban area, 10 km; outskirts, 50 km	100 bps	Terminal device to front-end application
LoRaWAN	IBM, CISCO	2015	250,000	ISM band, sub-1 GHz	Urban area, 3–5 km; outskirts, 15 km	300 bps–50 kbps	Communication protocol
Weightless	ARM, NEUL	2015	1,000,000	ISM band, sub-1 GHz	5 km+(-N) 2 km+(-P)	30–100 kbps(-N) 100 kbps(-P)	Communication protocol
NB-IoT	3GPP	2016	100,000	GSM or LTE band	20 km	~50 kbps	Communication protocol

**Table 2:** Comparison between the proposed solution and other methods.

Method	Signal Repeat Station	External Battery	Using NB-IoT in Areas with 3G/4G Telecommunication Signal	Consideration of Heavy Rain
Reference [43]	No	No	No	No
Reference [44]	No	No	No	No
Reference [45]	No	No	No	No
Reference [46]	No	No	No	No
Reference [47]	No	No	No	No
Reference [48]	No	No	No	No
Reference [49]	Yes	No	Yes	No
Reference [50]	No	No	No	No
Reference [51]	No	No	No	No
Reference [52]	No	No	No	No
The proposed method	Yes	Yes	Yes	Yes

**Table 3:** Correspondence between distance (*d*) and signal strength using the average RSSI from the LoRa-support solar micro-weather station on a mountain slope without rain.

Distance (km)	RSSI on Mountain Slope 1	RSSI on Mountain Slope 2	RSSI on Mountain Slope 3	RSSI on Mountain Slope 4
0.1	−64 dBm	−63 dBm	−62 dBm	−63 dBm
0.2	−68 dBm	−70 dBm	−67 dBm	−69 dBm
0.3	−74 dBm	−75 dBm	−71 dBm	−72 dBm
0.4	−83 dBm	−83 dBm	−80 dBm	−78 dBm
0.5	−104 dBm	−102 dBm	−103 dBm	−101 dBm
0.6	−122 dBm	−121 dBm	−122 dBm	−120 dBm

**Table 4:** Correspondence between distance (*d*) and signal strength using the average RSSI from the LoRa-support solar micro-weather station on the mountain slope in the case of heavy rain.

Distance (km)	RSSI on Mountain Slope 1	RSSI on Mountain Slope 2	RSSI on Mountain Slope 3	RSSI on Mountain Slope 4
0.1	−77 dBm	−78 dBm	−76 dBm	−75 dBm
0.2	−81 dBm	−80 dBm	−79 dBm	−81 dBm
0.3	−90 dBm	−89 dBm	−87 dBm	−89 dBm
0.4	−98 dBm	−96 dBm	−97 dBm	−98 dBm
0.5	−118 dBm	−120 dBm	−121 dBm	−119 dBm
0.6	−132 dBm	−131 dBm	−132 dBm	−130 dBm

**Table 5:** Correspondence between distance (*d*) and signal strength using the average RSSI from the LoRa-support solar micro-weather station at the mountain edge without rain.

Distance (km)	RSSI at Mountain Edge 1	RSSI at Mountain Edge 2	RSSI at Mountain Edge 3
0.6	−63 dBm	−67 dBm	−64 dBm
0.7	−70 dBm	−70 dBm	−69 dBm
0.8	−73 dBm	−75 dBm	−74 dBm
0.9	−78 dBm	−80 dBm	−79 dBm
1.0	−80 dBm	−79 dBm	−83 dBm
1.1	−96 dBm	−95 dBm	−93 dBm
1.2	−121 dBm	−122 dBm	−120 dBm
1.3	−129 dBm	−130 dBm	−131 dBm

## Data Availability

Not applicable.
